# Mutation in the Arabidopsis *regulatory-associated protein TOR 1B* (*RAPTOR1B*) leads to decreased jasmonates levels in leaf tissue

**DOI:** 10.1080/15592324.2019.1649567

**Published:** 2019-08-05

**Authors:** Mohamed A. Salem, Patrick Giavalisco

**Affiliations:** a Department of Pharmacognosy, Faculty of Pharmacy, Menoufia University, Shibin Elkom, Egypt; b Max Planck Institute of Molecular Plant Physiology, Potsdam-Golm, Germany; c Max Planck Institute for Biology of Ageing, Cologne, Germany

**Keywords:** Arabidopsis, TOR, RAPTOR, jasmonates, arabidopsides, wounding, JA, OPDA

## Abstract

The Target of Rapamycin (TOR) complex (TORC) regulates plant growth and development by modulation of metabolism in response to environmental cues. TORC contains in its core the TOR kinase and two interacting partners, namely; regulatory-associated partner of TOR (RAPTOR) and lethal with sec thirteen protein 8 (LST8). RAPTOR is described to act as a scaffold protein which recruits substrates for phosphorylation to the TOR kinase.

In the current manuscript we show that mutation of Arabidopsis *RAPTOR1B* leads to significantly decreased levels of free jasmonic acid (JA), jasmonoyl-(*L*)-isoleucine (JA-Ile) as well as its biosynthetic precursor 12-*oxo*-phytodienoic acid (OPDA). Although *raptor1b* leaves showed decreased basic JA level compared to WT, the mutant responded substantially to wounding stress by producing the same amount of JA as WT. Furthermore, we could show that the chemical inhibition of TOR by AZD-8055 led to an opposite response. AZD-treated WT and *raptor1b* leaves accumulated high JA levels. These results strongly imply that the TOR signaling pathway is responding differentially to the inhibition of the TOR kinase as compared to the inhibition of the scaffold protein RAPTOR.

## Overview

TOR is an atypical Ser/Thr protein kinase that is conserved among all eukaryotes.^1,2^ The TOR kinase has been shown to be crucial for plant growth and metabolism.^3^ TOR has can assemble into at least two distinct protein complexes in most organisms called TOR complex 1 and 2 (TORC1 and TORC2)^4^ However, plants seem to only contain the core components of TORC1, namely RAPTOR and LST8.^5,6^ In contrast to the embryo-lethal *tor*-mutant lines,^7^ *raptor1b* (At3g08850) and *lst8-1* (At3g18140) mutants are viable but show significant growth and developmental phenotypes.^5,6,8,9^

In several, previous publications it has been shown that TOR regulates growth, development and the plant’s life span, by modulating transcription, translation and metabolism.^5,10–16^ Additionally, the glucose-activated TOR signaling regulates many genes involved in cell proliferation, cell cycle, nucleotide synthesis and meristematic growth.^14,16^

Plant hormones have been shown to regulate crucial aspects of plant growth and development by modulation of metabolism in response to environmental conditions.^17–20^ TOR plays a critical role in regulating phytohormone signaling pathways in Arabidopsis during growth and development.^21–25^ For instance, auxin-activated TOR signaling orchestrates plant growth and development.^26–28^ Moreover, AZD-induced TOR inhibition and mutation of *RAPTOR1B* or *LST8-1* led to significantly decreased abscisic acid (ABA) level in young seedlings.^8^ Contrary to this report, we have found that *raptor1b* accumulate high amounts of ABA in their seeds.^9^

We have shown recently that *raptor1b* plants show growth retardation and developmental delay phenotypes,^9,29^ suggesting severe imbalances in the phytohormonal equilibrium. In recent years, it was shown that complex cross talk between various plant hormones is essential for the adjustment of plant growth and development in response to environmental conditions.^19,20^ As has been shown previously, *raptor1b* showed accumulation of JA and OPDA in their dry seeds and this was strongly associated with a delayed-germination phenotype.^9^ Oxylipins area class of lipid-derived metabolites that function as regulators in plant response to stresses.^30^ Oxylipins include jasmonates like JA, its active form JA-Ile and the JA precursor *cis*-OPDA.^31,32^ Jasmonates are known to be involved in the regulation of plant growth and development, root growth, reproduction, trichome development, senescence as well as plant defense against biotic and abiotic stresses.^30–33^ Arabidopsides are complex galactolipids that contain esterified OPDA or dinor-OPDA (dn-OPDA) and they were described as storage sources of OPDAs.^30–32^

In this report, we show that jasmonate levels are not only differential in RAPTOR-deficient source- (leaf) and sink- (seed) tissues, but that they are also differentially regulated during the day/night cycle. Accordingly, mutation of *RAPTOR1B* led to significantly increased levels of JA, JA-Ile and OPDA in the seeds, while the levels were decreased in leaf tissue. We also found that *raptor1b* leaves, which have reduced jasmonate levels, accumulated comparable jasmonate levels as WT, when subjected to wounding stress. These results indicate that the wounding response is, contrary to the steady-state levels, independent of a functional TORC1. Beyond these RAPTOR1B-specific results, we further show that hormonal control through TORC1 can have quite different outcomes depending on the selected complex component. While the KO of RAPTOR1B leads to substantial decrease of JA levels, the inhibition of the TOR kinase activity shows opposing hormonal phenotypes.

## Differential control of jasmonate levels in source and sink tissues of *raptor1b* mutants

**Figure 1.**
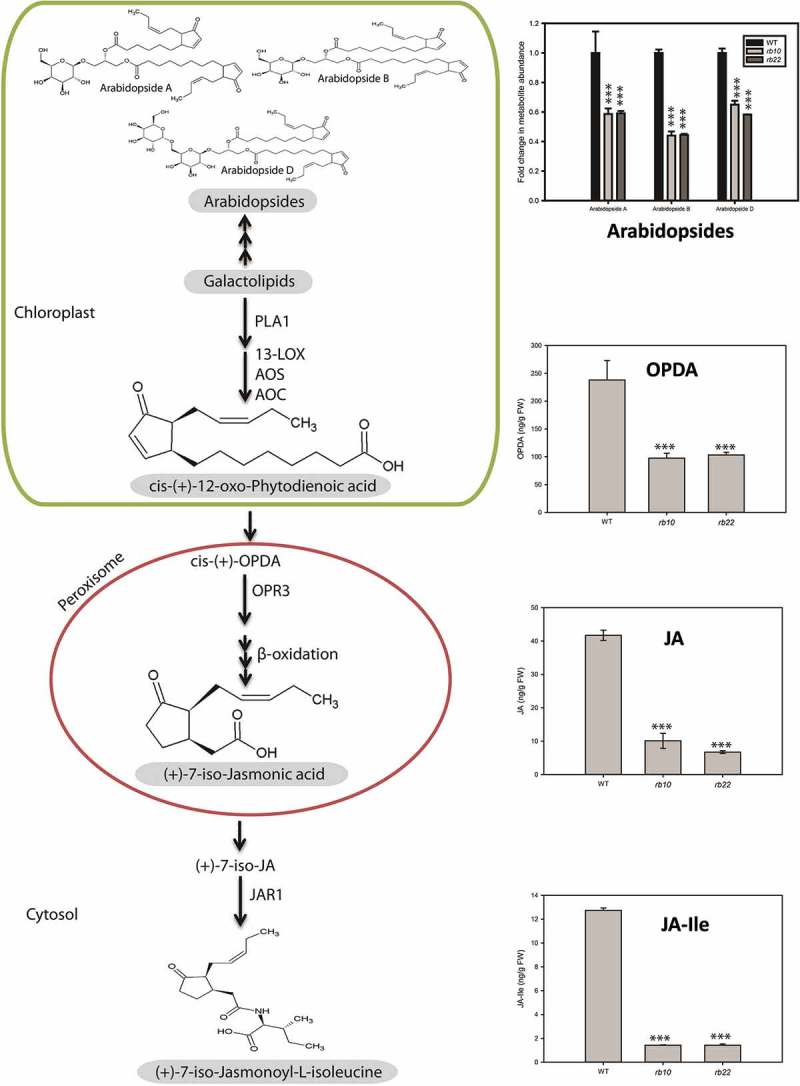
Influence of *RAPTOR1B* mutation on the endogenous levels of leaf jasmonates. *raptor1b* leaves show significant decrease in levels Arabidopsides (A, B and D), 12-oxo-phytodienoic acid (OPDA), free jasmonic acid (JA) and jasmonoyl-(*L*)-isoleucine (JA-Ile). For extraction, the rosettes of *raptor1b* and WT were harvested at the same developmental stage (10 rosette leaves) from plants cultivated on soil under normal light LD growth conditions. Error bars indicate mean ± SD for five biological replicates. Black asterisks indicate significantly different from the WT under the same condition (***P< .001, Student’s *t*-test).

JA and its precursor OPDA are known to regulate different aspects of plant growth and they have been shown to inhibit seed germination.^30,33–35^ We recently reported increased levels of JA and OPDA and delayed germination in *raptor1b* mutated seeds.^9^ To further investigate the cross talk between RAPTOR1B and JA in vegetative tissue, we measured jasmonate levels in developmentally matched (10-rosette leaf stage)^36^ WT and *raptor1b* leaves. The results showed that the endogenous jasmonate concentrations of OPDA, JA-Ile and free JA were significantly decreased in *raptor1b* leaves (Figure 1). Concomitantly, galactolipids containing OPDA or dinor-OPDA, so-called Arabidopsides,^37^ were also significantly decreased in *raptor1b* mutated leaves (Figure 1).

**Figure 2.**
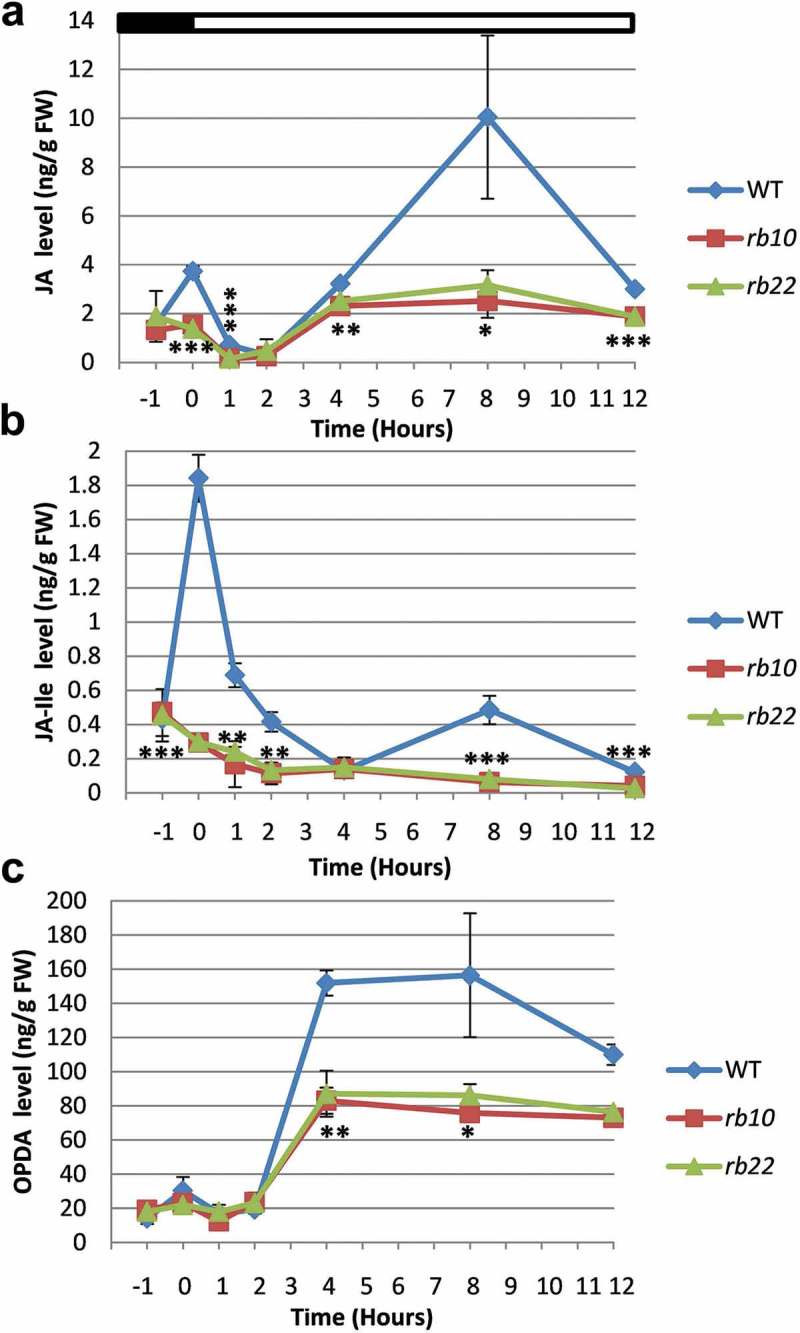
Diurnal changes of jasmonates in WT and *raptor1b* leaves. The first time point (−1) represents samples that were harvested 1 h before illumination while 0 h represents samples harvested just before illumination. The time points of 1, 2, 4, 8 and 12 h represent samples that were harvested in the light period after 1, 2, 4, 8 and 12 h after illumination, respectively. Error bars indicate mean ± SD for five biological replicates. Black asterisks indicate significant difference between WT and *raptor1b* under the same condition (*P< .05, **P< .01,***P< .001, Student’s *t*-test). White and black bars on the top of graphs indicate light and dark, respectively. FW are given to fresh weight.

To further investigate if the reduced jasmonates levels of *raptor1b* were light-dependent, we measured the JA, JA-Ile and OPDA levels of samples that were harvested before and during illumination. WT leaves showed significant changes in jasmonate levels during the light/dark transition and throughout the daily light period. Accordingly, the levels of JA, JA-Ile showed two distinct concentration maxima, one immediately before the beginning of the light phase and at the second, even more pronounced one, in the middle of the light period (Figure 2A and B). Differential to the bimodal curve shape of JA and JA-Ile, OPDA showed a steep increase between the second and third hour of light, which is maintained until the middle of the day, where the concentrations steadily decline (Figure 2C). As a consequence, the absolute concentration of OPDA in *raptor1b* leaves reaches only 50% of the WT (Figure 2C). Even more differential than the OPDA levels were the JA and JA-Ile levels, which were not only decreased at most analyzed time points, but also lacked the two concentration maxima immediately before and in the middle of the light phase (Figure 2A and B).

## *raptor1b* mutants can induce the synthesis of jasmonates in leaf tissue after wounding

Based on the observation of the reduced jasmonates levels in leaf tissue, we asked if the *raptor1b* mutation might negatively control jasmonate concentration at the basic synthesis level. To address this question, we subjected WT and *raptor1b* leaves to wounding, an abiotic stress known to induce jasmonate production.^33,38^ Leaves were harvested from control and treated plants at 0.5, 1, 2, 4 and 8 h after wounding.

**Figure 3.**
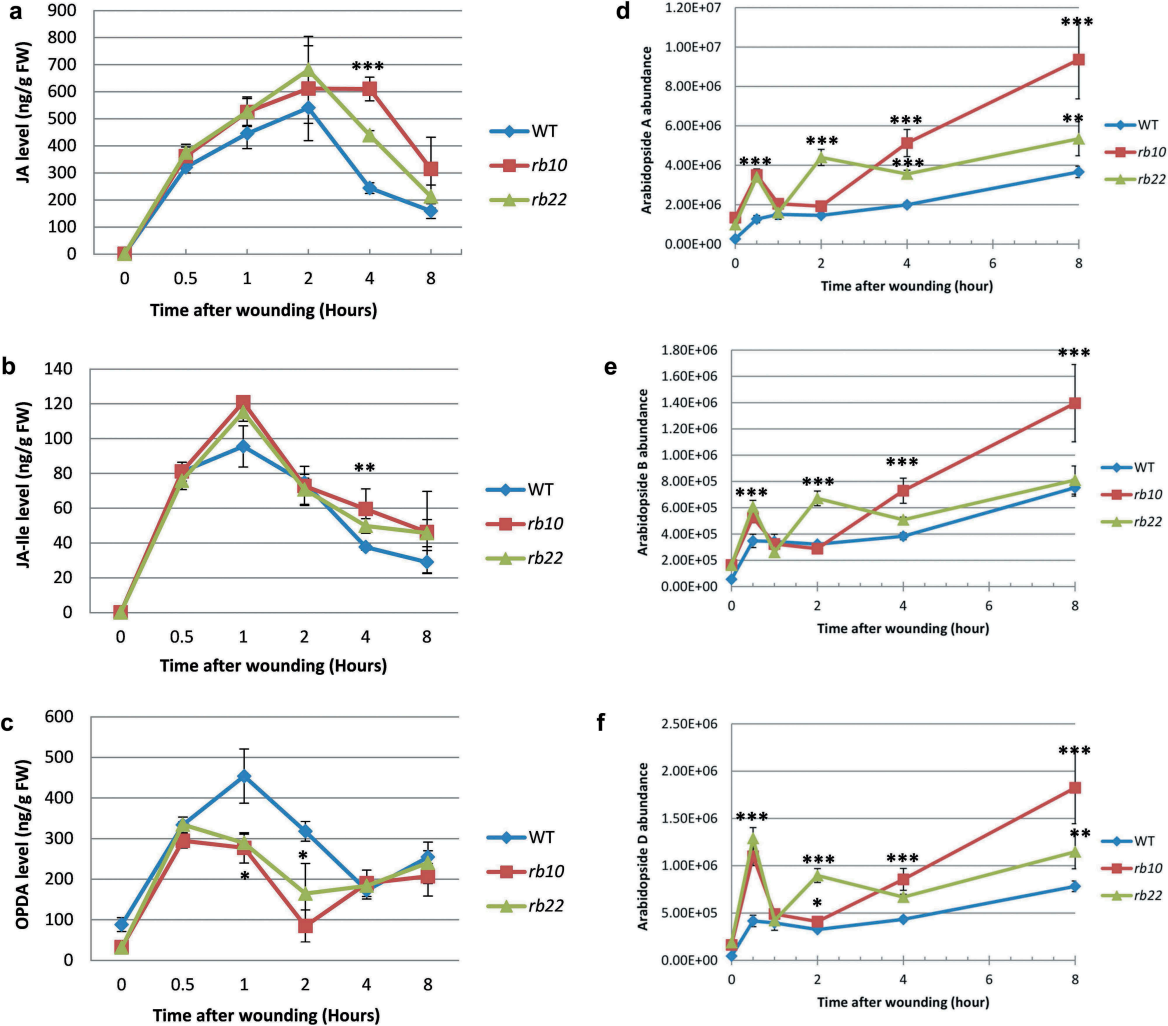
Wounding-induced changes of jasmonates and arabidopsides in WT and *raptor1b* leaves. *raptor1b* leaves showed a significant increase in endogenous JA (A) and JA-Ile (B) levels after 4 h of wounding and a significant decrease in endogenous OPDA level (C) after 1–2 h of wounding. *raptor1b* responded to wounding stress by having high Arabidopsides (D-F). For wounding treatment, samples were harvested at 0, 0.5, 1, 2, 4 and 8 h after mechanical wounding. Error bars indicate mean ± SD for five biological replicates. Black asterisks indicate significant difference between WT and *raptor1b* under the same condition (*P< .05, **P< .01,***P< .001, Student’s *t*-test).

*raptor1b* plants showed almost identical response curves as WT leaves for JA and JA-Ile, still reaching partially higher absolute levels 4 h after the wounding stress (Figure 3A and B). Contrary to JA and JA-Ile, their precursor, namely OPDA, was unable to follow the steep increase seen in the WT response curve. While WT OPDA levels increased the first 2 h after wounding, the levels in *raptor1b* decline already after 0.5 h and reached their minimum after 2 h of wounding (Figure 3C). However, OPDA returned to WT levels at 4 and 8 h after wounding (Figure 3C).

To further investigate this differential OPDA-effect after wounding treatment, we compared the levels of the major Arabidopsides,^37^ which can function as a sink of synthesized OPDA and dnOPDA, after wounding. Surprisingly, *raptor1b* leaves accumulated more than their respective WT at almost all measured time points (Figure 3D–F), indicating that the synthesized OPDA is either directly esterified to the galactolipids or the acyl fatty acids of the galactolipids are oxidized to oxylipins on the galactolipid. The second possibility seems to be more likely, since wounding stress has been shown to activate phospholipases, inducing oxidation of fatty acids on galactolipids.^39^

## Chemical and genetic inhibition of TOR affects jasmonates differently

A recent report showed that inhibition of TOR using the TOR active-site inhibitor AZD-8055, led to significantly increased JA level in 2-week-old Arabidopsis seedlings, grown on agar plates.^24^ Our results from *raptor1b* plants seem to contradict this report, since the genetic mutation of *RAPTOR1B* led to decreased jasmonate level. One possible explanation for the observed difference may be the developmental stage that was used for JA measurement. In our study, we used vegetative leaves of plants at the10-rosette leaf stages (25 days after imbibition, DAI)^36^ grown on soil, while 2-week-old seedlings, grown on ½ MS medium plates, possibly containing sugar, were used in the previous study.^24^ We hypothesized that JA level is different between the genetic TOR inhibition by *RAPTOR1B* mutation used in this study and the chemical TOR inhibition used previously.^24^ To address these points in details, we decided to quantify jasmonate level in young Arabidopsis seedlings using either AZD-induced chemical TOR inhibition, genetic mutation of *RAPTOR1B* or combination of both.

**Figure 4.**
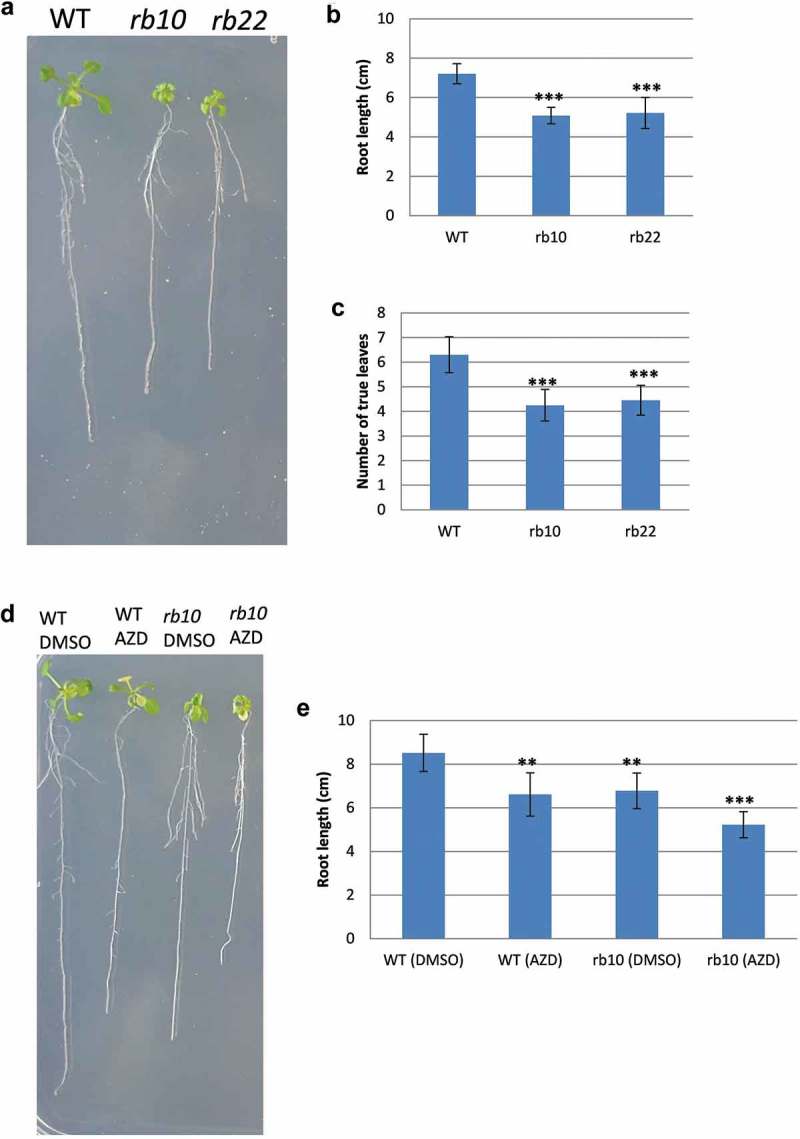
Influence of *RAPTOR1B* mutation and AZD treatment on Arabidopsis growth. (A) 17-days-old Arabidopsis seedlings of WT and *raptor1b*. (B) and (C) Influence of *RAPTOR1B* mutation on the root length (B) and number of leaves (C). (D) 17-days-old Arabidopsis seedlings of WT and *raptor1b* after treatment with 1 µM AZD in DMSO for 3 days. (E) Influence of AZD treatment on the root length of WT and *raptor1b*. Seedlings were grown on 0.5x MS plates containing 1% agar. After 14 days, WT and *raptor1b* seedlings were transferred to new plates supplemented with 1 µM AZD in DMSO. Control non-treated WT and *raptor1b* were transferred to DMSO-supplemented plates. Error bars indicate mean ± SD for five biological replicates. Black asterisks indicate significant difference between WT, AZD-treated WT and *raptor1b* under the same condition (**P< .01, ***P< .001, Student’s *t*-test).

*raptor1b* seedlings grown on ½ MS plates showed significant decrease in the primary root length and number of rosette leaves (Figure 4A–C), consistent with previous data.^8,9^ This reduction was more obvious when 2-week-old seedling of *raptor1b* was treated for 3 days with 1 µM AZD (Figure 4D and E). Arabidopsis WT seedlings showed also significant reduction in the primary root length upon AZD treatment (Figure 4E), consistent with previous reports.^8,24^

**Figure 5.**
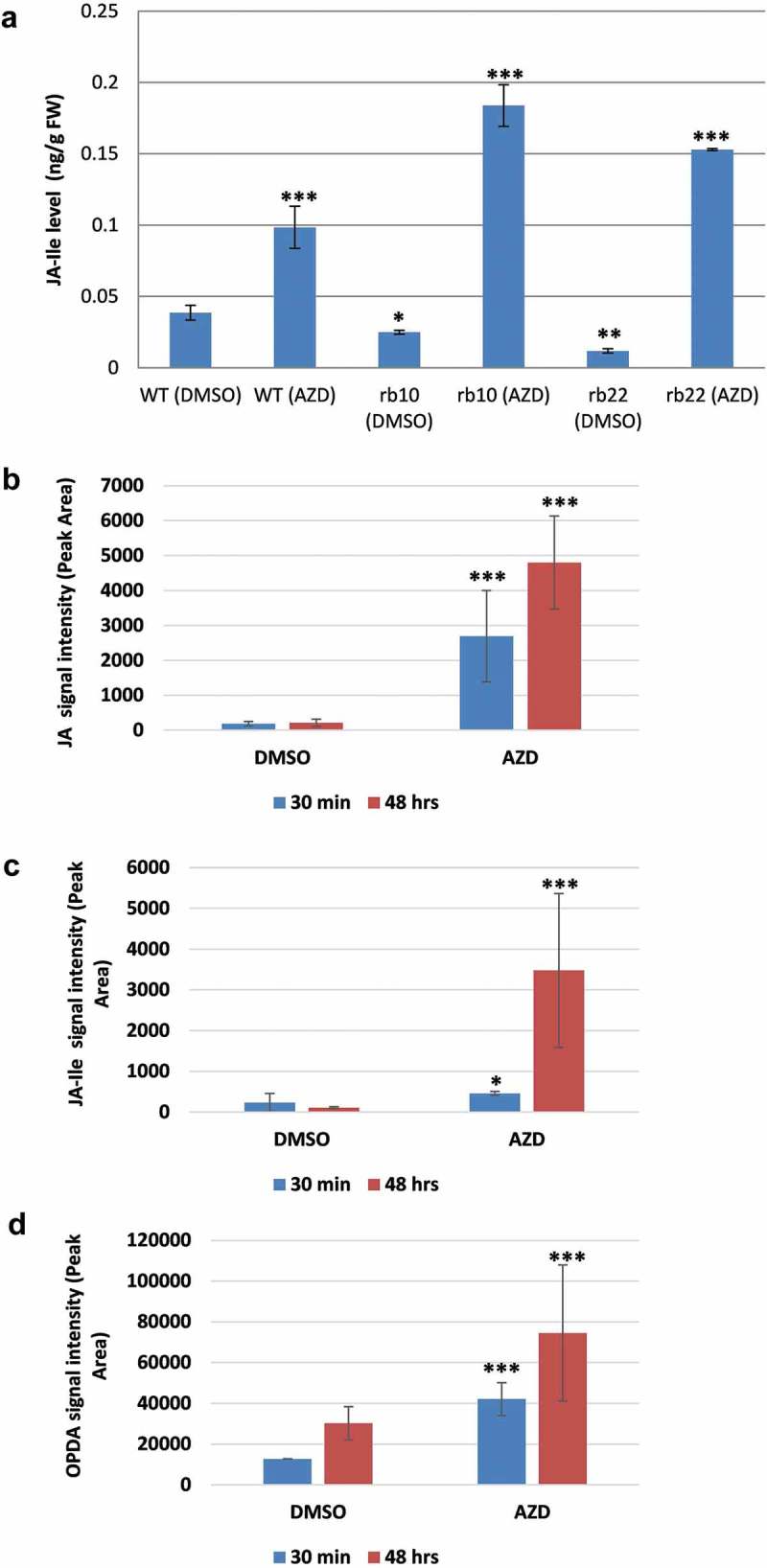
AZD treatment is associated with significant increase in jasmonates level in Arabidopsis. AZD-induced TOR inhibition leads to significant increase in jasmonoyl-*L*-isoleucine (JA-Ile) level in 17-days-old seedlings of WT and *raptor1b* seedlings (A). WT showed a significant increase in endogenous JA (B), JA-Ile (C) and OPDA (D) levels after 30 min and 48 h of AZD treatment of Arabidopsis WT growing in seedling cultures.17-days-old seedlings of WT and *raptor1b* seedlings grown on 0.5x MS agar were harvested after 3 days of treatment with 1 µM AZD in DMSO. DMSO-treated WT and *raptor1b* mutants were used as control. Liquid seedling cultures^40^ of WT Arabidopsis were supplemented with 0.5x MS solution containing 15 mM glucose for 1 week. For chemical inhibition of TOR, 7-day-old seedlings were supplemented with 0.3 µM AZD in DMSO. Control WT seedlings were supplemented with DMSO. Error bars indicate mean ± SE for five biological replicates. Black asterisks indicate significance difference (*P< .05,**P< .01, ***P< .001, Student’s *t*-test).

To further investigate the jasmonate levels in this system, young (17-day-old) *raptor1b* seedlings were harvested together with their respective WTs. The results of the JA analysis showed a significant decrease in JA-Ile level in *raptor1b* (Figure 5A), consistent with the reduced levels in vegetative leaves (Figure 1). Surprisingly, when WT seedlings were treated for 3 days with 1 µM AZD, they showed significant accumulation of JA-Ile (Figure 5A). This accumulation was even more abundant for *raptor1b* that showed more than sevenfold increase in JA-Ile level upon AZD treatment (Figure 5A). To exclude the possibility that this JA-Ile increase was induced by mechanical force during the transfer of seedlings from agar plates to AZD-supplemented plates, we repeated the AZD treatment using Arabidopsis liquid seedling cultures.^40^ For this purpose, liquid seedling cultures were incubated in medium with slightly lower AZD concentration (0.3 µM) as compared to the plate experiment (1 µM). Consistent with results obtained from the plate experiment, WT showed a significant increase in endogenous JA, JA-Ile and OPDA levels after short term (30 min) and long term (48 h) AZD treatment (Figure 5B–D).

## Conclusion and perspectives

In the present study, *raptor1b* showed significant reduction in the levels of the jasmonates as well as their precursors. Furthermore, *raptor1b* leaves, when subjected to wounding, the well-known mechanical abiotic stress that induces jasmonates, responded in the same way as the WT control. Additionally, AZD-induced TOR inhibition or combination of *RAPTOR1B* mutation and AZD treatmentinduced jasmonate production, similar to the wound-induced stress response. RAPTOR1B, a conserved TOR-interactor, is a scaffold protein which recruits substrates for the TOR kinase and *raptor1b* mutants showed the molecular and physiological phenotypes of genetic or rapamycin-induced TOR inhibition.^29^ AZD-8055 belongs to the new generation of ATP-competitive inhibitors, which directly target the ATP-binding site of the TOR kinase in both TOR complexes (TORC1 and TORC2).^41–43^ Because the Arabidopsis TOR kinase domain shares high amino acid sequence similarity with the human TOR kinase domain, AZD-8055 was accepted by the plant community as a promising tool for investigating TOR functions in plants.^44^ Previous studies have shown that different ATP competitive inhibitors of TOR can cause the very generic phenotype of plant growth inhibition.^21,24,45–48^ However in animal cells, it was shown that rapamycin and Torin 1 (a first-generation ATP-competitive inhibitor) induce different cellular and molecular responses.^49^ Because the specific components of TORC2 seem to be absent in photosynthetic organisms, there is no evidence for the existence of a plant TORC2. However, an equivalent complex may exist in plants containing functionally consistent but sequence-wise diverse properties, as compared to the proteins of the yeast and mammalian counterparts.^44^ Therefore, we assume that the chemical inhibition induced by AZD could lead to the inhibition of TORC but possibly also induces other stress responses which are TOR-independent (off-targets). Another, less likely possibility could be that plants may have a second TOR complex, which negatively regulates jasmonates, which is RAPTOR1B- independent. Both hypotheses would need further analysis, but are urgently required to avoid misleading interpretations of contradicting results.

Using rice as a model system (*Oryza sativa* L. cultivar Kitaake), De Vleesschauwer et al. showed that TOR antagonizes the action of JA in leaves.^50^ The authors showed that significant upregulation of JA-responsive genes has been detected in *TOR* and *Raptor* RNAi plants as well as rapamycin-treated WT, Whereas, TOR overexpression resulted in significant downregulation of JA marker genes as well as significant reduction in the endogenous JA content level relative to WT.^50^ Additionally, *Raptor* RNAi resulted in significant accumulation of JA.^50^ This contradicting observation could indicate that there is a functional difference in RAPTOR activity between monocots and dicots. Such difference has been also detected before in rice where repression of *RAPTOR* led to significant reduction in photosynthetic efficiency, while this phenotype has not been observed in Arabidopsis.^29,51^ Taken together, the present study confirms that RAPTOR1B positively regulates jasmonates; however, further molecular and genetic studies are necessary to obtain a deeper understanding of this partially differential regulation.
